# Does the use of telephone reminders to increase survey response rates affect outcome estimates? An ancillary analysis of a prospective cohort study of patients with low back pain

**DOI:** 10.1186/s12891-021-04787-4

**Published:** 2021-10-20

**Authors:** Christina Lyngsø Udby, Allan Riis, Janus Laust Thomsen, Nanna Rolving

**Affiliations:** 1 Department of Public Health, Aarhus University, Nordre Ringvej 1, 8000 Aarhus, Denmark; 2 Department of Physiotherapy and Occupational Therapy, Aalborg University Hospital, Hobrovej 18–22, 9000 Aalborg, Denmark; 3 Department of Physiotherapy, University College of Northern Denmark, Selma Lagerløfs Vej 2, 9220 Aalborg Ø, Denmark; 4 Center for General Practice at Aalborg University, Fyrkildevej 7, 9220 Aalborg Ø, Denmark; 5 Diagnostic Centre, Silkeborg Regional Hospital, Falkevej 1–3, 8600 Silkeborg, Denmark; 6 DEFACTUM, Central Denmark Region, Denmark

**Keywords:** Research design, Reminder systems, Patient outcome assessment, Low Back pain

## Abstract

**Background:**

Research is often undertaken using patient-reported outcomes from questionnaires. Achieving a high response rate demands expensive and time-consuming methods like telephone reminders. However, it is unknown whether telephone reminders change outcome estimates or only affect the response rate in research of populations with low back pain (LBP). The aim is to compare baseline characteristics and the change in outcome between patients responding before and after receiving a telephone reminder.

**Methods:**

This is an ancillary analysis of data from a prospective cohort study employing questionnaires from 812 adults with LBP lasting more than 3 months. Patients not responding to the 52-week questionnaire were sent reminder emails after two and 3 weeks and delivered postal reminders after 4 weeks. Patients still not responding were contacted by telephone, with a maximum of two attempts. Patients were categorised into three groups: 1) patients responding before a telephone reminder was performed; 2) patients responding after the telephone reminder and 3) patients not responding at all. A positive outcome was defined as a 30% improvement on the Roland Morris Disability Questionnaire after 52 weeks.

**Results:**

A total of 695 patients (85.2%) responded. Of these, 643 patients were classified in Group 1 and 52 patients were classified in Group 2. One hundred seventeen were classified in Group 3. No differences in outcome or baseline characteristics was found. In Group 1, 41.3% had a positive outcome, and in Group 2 48.9% had a positive outcome (*P* = 0.297). In group 3, non-respondents were younger, more often unemployed, more often smokers, more often reported co-morbidity, and reported higher depression scores than respondents.

**Conclusions:**

Using a telephone reminder had no consequence on outcome estimates nor were there any differences in baseline characteristics between patients who responded before or after the telephone reminder.

**Trial registration:**

The initial trial was registered in Clinicaltrials.gov (NCT03058315).

## Background

Low back pain (LBP) is a major global challenge, and the burden of back-related disability is expanding with the increasing ageing population, placing LBP as the leading worldwide cause of years lived with disability [1]. LBP-related costs associated with work disability and attributed to health care is massive, with the societal costs for back pain being estimated to 1 to 2% of the gross national product [2–4]. The majority of these costs (80–90%) is caused by productivity loss due to work-disability [5]. It has recently been emphasised that intensified research efforts and global initiatives are needed to address the burden of LBP as a public health problem [5]. Research within public health is often undertaken using patient-reported outcomes from questionnaires to explore a potential association between a given exposure and an outcome. However, the strength of data derived from questionnaires depends on the level of response rates [6]. Consequently, the response rate is considered a central indicator of data quality, with regard to internal validity. Furthermore, poor response rates reduce the statistical power of the study [7]. Non-response may introduce bias if the responders are different from non-responders in terms of baseline characteristics [8]. Consequently, achieving adequate response rates is assumed to reduce the risk of bias and increase internal validity [9].

Reducing questionnaire length, providing incentives or using reminders have been suggested for improving response rates [10–13]. However, achieving a high response rate by employing multiple reminders is often expensive and time-consuming [14, 15]. Previous studies have found that prevalence estimates and exposure-outcome relationships may not be influenced by an increase in response rate [16–19]. Telephone reminders might be a stronger method to increase the response rate. However, compared to postal or e-mail reminders, telephone reminders are more time-consuming for researchers [10]. To our knowledge, the consequences of employing telephone reminders in survey research with patients with LBP has not previously been investigated. However, we hypothesised that patients who responded after telephone reminders had worse symptoms at baseline and were less likely to have a positive outcome. The aim is to compare baseline characteristics and the change in outcome between patients responding before and after receiving a telephone reminder.

## Methods

This study is reported according to the STROBE guidelines for observational studies in epidemiology [20].

### Study design

This is an ancillary analysis of data from a prospective cohort study with follow-up of 1 year, using questionnaire data retrieved through an electronic survey [21].

### Participants and setting

The data derive from a consecutive series of adults with LBP, referred from general practice to the regional Spine Centre at Silkeborg Regional Hospital in Denmark. There was a follow-up time of 52 weeks [19]. To be referred, patients had to report LBP of at least 3 months’ duration, been offered conservative treatment and have received an MRI prior to the consultation. To be eligible to the study patients had to fulfil the following criteria.

#### Inclusion criteria

1. ≥18 years of age
2. LBP was the primary cause of the referral to the Spine Centre

#### Exclusion criteria

1. Known spinal fractures, inflammatory disease or infection
2. Suspected malignancy
3. Missing information about the number of reminders used (*n* = 4)

**Fig. 1:**
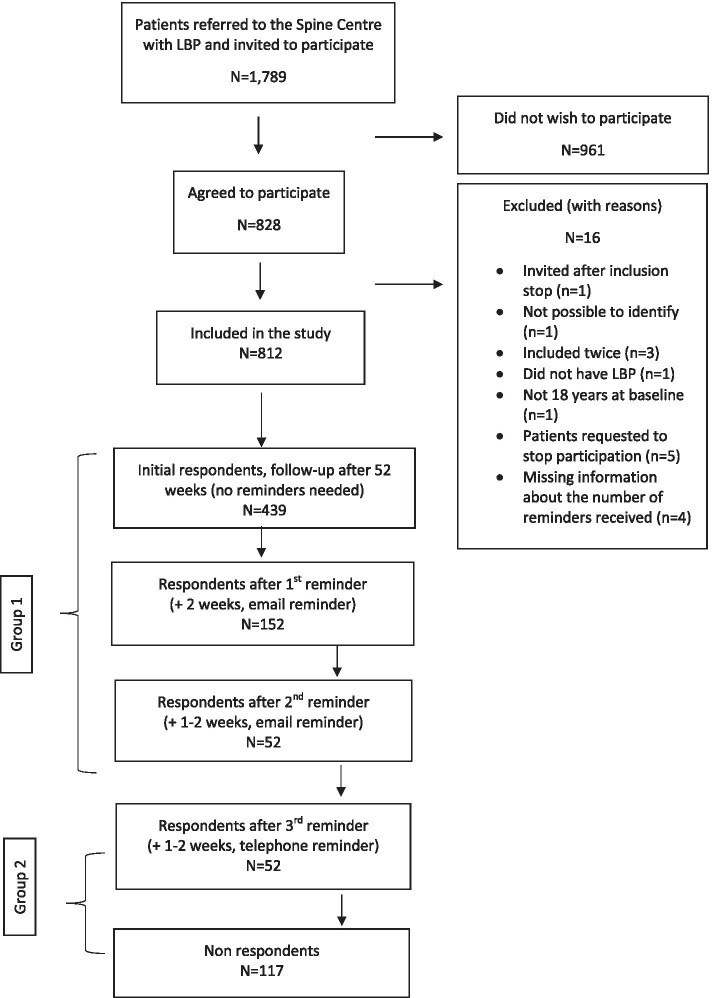
Flowchart of patient participation and data collection. *NOTE:* 812 patients with low back pain referred from general practice to the regional Spine Centre at Silkeborg Regional Hospital in Denmark were included in the study. Six hundred forty-three responded without needing a telephone reminder and 52 responded after receiving a telephone reminder

Patients referred to the Spine Centre routinely receive a digital letter with a link to a standard, online questionnaire regarding their symptoms. For all patients fulfilling the inclusion criteria and not the exclusion criteria, an extra page became visible at the end of the questionnaire, informing them about the study. Consenting patients were then requested to reply to extra questions in addition to the standard questionnaire and to complete a follow-up questionnaire after 52 weeks. Both baseline and follow-up questionnaires were electronic questionnaires to be completed online, using a link in the invitation letter. Patients not responding to the 52-week questionnaire were sent reminder emails after two and 3 weeks and delivered postal reminders after 4 weeks. Patients who still did not respond were contacted by telephone, with a maximum of two attempts. When patients did not pick up, a message was left on the answering machine if this was possible. Text messaging was not used in this study. Patient participation and data collection is illustrated in Fig. 1. Patients were considered respondents if they returned the questionnaire, regardless of completeness.

### Explanatory variables

Patients were categorised into two groups according to their response pattern. In addition, a third group was defined, consisting of patients who did not respond to any of the follow-up procedures (Group 3). This allows for comparison between baseline variables of all respondents (Group 1 + 2) and patients who did not respond despite the reminder procedures:

- Group 1: ‘non-telephone reminder respondents’ consisted of patients who initially responded after an automatically generated e-mail was sent approximately 52 weeks after their first visit to the Spine Centre, and patients who responded to either e-mail reminders or postal reminder. .
- Group 2: ‘telephone reminder respondents’ consisted of patients who responded to a telephone call (following non-response to the previous reminders).
- Group 3: Patients who did not respond to any reminders were classified as ‘non-respondents’.

### Outcome

The Roland Morris Disability Questionnaire (RMDQ) was the outcome measure. The outcome was dichotomised by considering patients reporting a 30% or higher improvement after 52 weeks a success. The RMDQ is a widely used patient-reported outcome questionnaire designed to measure self-rated disability due to back or leg pain [22, 23]. The version used in this study consists of 23 items with ‘yes’ or ‘no’ response options and greater levels of disability are reflected by higher numbers on a 23-point scale [24]. The RMDQ is validated in Danish and its use is recommended in clinical practice as well as in research [25]. A 30% improvement in the RMDQ from baseline values has previously been proposed as a minimal important change [26, 27].

### Pilot testing of questionnaires

The questionnaire has been routinely administered at the Spine Centre since January 2016. However, since additional questions were included the complete questionnaire was pilot tested for face validity on 10 patients referred to the Spine Centre prior to this study [19]. This testing process involved the patients completing the questionnaire with one of the researchers (NR) present to observe and offer verbal feedback. This feedback led to minor question modifications. The RMDQ and other validated questionnaires remained unchanged [19].

### Sample size

The complete cohort (*N* = 816) from the original study was used [19]. However, patients with missing data on the administered reminder system were excluded from this study.

### Statistical analyses

Descriptive statistics are presented as total numbers (N) and proportions (%), as well as mean and standard deviation (SD) if the values were normally distributed. Otherwise, median and interquartile range (IQR), i.e. the 25th–75th percentile, is used.

The study aims to investigate whether changes in outcome estimates and baseline characteristics of patients with low back pain differ between patients responding to email reminders (group 1) and patients requiring telephone-reminders (group 2), and furthermore, to assess to what extent responders (group 1 + 2) differ from patients who do not respond at all (group 3). Fisher’s exact test was used to investigate differences in the proportion of patients who had achieved a clinically relevant improvement from baseline to follow-up (RMDQ improvement > 30%) between Group 1 and 2.

Fisher’s exact test was also used to investigate between-group differences in sex, level of education, employment status, sick leave, smoking status, history of lower back surgery, co-morbidity and pain status. A 2-sided t-test was conducted to investigate whether normally distributed continuous variables (age, RMDQ and LBPRS) differed between groups. A Wilcoxon rank-sum test was used to investigate whether non-parametric continuous variables (Major Depression Inventory score (MDI-10)) differed between Group 1 and 2.

Statistical analysis was performed using Stata/SE v.16.1 (StataCorp) and results were considered significant when *P* ≤ 0.05.

## Results

Between 1 April and 22 December 2017, 1789 patients with LBP were invited to participate in the primary study and 828 consented, of which 812 fulfilled the criteria for this study and were included. The average follow-up time was 52 weeks. A total of 643 patients returned the questionnaire without needing a telephone reminder and were classified in Group 1 as ‘non-telephone reminder respondents’. Fifty-two patients returned the questionnaire after they were reminded by telephone and were classified in Group 2 as ‘telephone reminder respondents’. The remaining 117 patients did not reply to the follow-up questionnaire at all and were classified in Group 3 as ‘non-respondents’. The telephone reminder increased the response rate from 79.2 to 82.5% (Fig. 1).

Mean age at baseline was 53 years (sd 13.7) with 453 (55.8%) being women, and the mean RMDQ score at baseline was 14.0 (sd 4.9), indicating a moderate level of disability. There was no statistically significant difference in baseline characteristics between Group 1 and 2 (Table 1).

**Table 1:** Baseline characteristics of patients in groups 1, 2 and 3

Outcome variables	Groups 1 (***N*** = 643)	Group 2 (***N*** = 52)	Differences Group 1 versus Group 2	Group 3 (***N*** = 117)	Differences Group 1 + 2 versus Group 3
**Age, years (sd)**	54 (13.3)	53 (13.3)	*P* = 0.425^6^	46 (14.3)	*P* < 0.001^6^
**Sex, female (%)**	365 (56.8)	27 (51.9)	*P* = 0.561^7^	61 (52.1)	*P* = 0.421^7^
**College-level education or higher**^**1**^**, yes (%)**	225 (35)	18 (34.6)	*P* = 1.000^7^	43 (36.8)	*P* = 0.754^7^
**Employed, yes (%)**^**2**^	585 (91.1)	43 (84.3)	*P* = 0.129^7^	96 (82.1)	*P* = 0.009^7^
**Sick leave, yes (%)**	94 (14.6)	9 (17.3)	*P* = 0.548^7^	24 (20.5)	*P* = 0.130^7^
**Current smoker, yes (%)**	98 (15.2%)	8 (15.4%)	*P* = 1.000^7^	31 (26.5%)	*P* = 0.005^7^
**History of low back surgery, yes (%)**	80 (12.4)	5 (9.6)	*P* = 0.664^7^	13 (11.1)	*P* = 0.878^7^
**Self-reported co-morbidity (“very bothered from”), yes (%)**	105 (16.3)	11 (21.2)	*P* = 0.340^7^	29 (24.8)	*P* = 0.038^7^
**Roland Morris Disability Questionnaire**^**3**^**, (sd)**	14 (4.8)	13.5 (5.2)	*P* = 0.466^6^	13.9 (5.2)	*P* = 0.418^6^
**Chronic pain (> 12 weeks), yes (%)**	583 (90.7)	46 (88.5)	*P* = 0.621^7^	106 (90.6)	*P* = 1.000^7^
**Major Depression Inventory**^**4**^ **(IQR**^**5**^**)**	20 (10-36)	20 (10-36)	*P* = 0.789^8^	28 (14-52)	*P* < 0.001^8^
**Numerical Pain Rating (0–10), (sd)**	5.2 (2.5)	5.0 (2.3)	*P* = 0.714^6^	5.3 (2.4)	*P* = 0.517^6^

NOTE:

Group 1: patients responding to email reminders; Group 2: patients responding to telephone reminders; Group 3: Patients not responding. ^1^College level education equals bachelor level. ^2^2 missing.^3^Roland Morris Disability Questionnaire (0–23 points, high score = high disability).^4^Scoring is reported using proportional recalculation (0–100, high score = more depressed).^5^IQR = interquartile range (25th; 75th percentile). ^6^Tested using 2-sided t-test.^7^Tested using Fisher’s exact test.^8^Tested using Wilcoxon rank-sum test

In terms of the proportion of patients achieving a clinically relevant improvement in functional ability (i.e. minimum 30% improvement on the RMDQ over 1 year), 257 (41.3%) had a clinically relevant improvement in RMDQ in Group 1 and 24 (48.9%) had a clinically relevant improvement in RMDQ in Group 2, *P* = 0.297. In group 1, 21 observations were missing and in Group 2, three observations were missing.

In comparison with patients responding to the follow-up questionnaire (Group 1 + 2), non-respondents (Group 3) were significantly younger (*P* < 0.001), more often unemployed (*P* = 0.009), more often smokers (*P* = 0.005), more frequently reported co-morbidity (*P* = 0.038) and had higher depression scores (*P* < 0.001) (Table 1).

## Discussion

### Principal findings

In the present study, employing a telephone reminder showed that patients who responded before or after the telephone reminder had no difference in baseline characteristics or outcome estimates. Patients who did not respond to either written or telephone reminders (Group 3) were significantly younger, more often unemployed, more often smokers, reported more co-morbidity and had higher depression scores than patients in the two response groups (Group 1 + 2).

### Response rate

The increase in response rate after employing a telephone reminder is in line with findings from a systematic review from 2009, where response rates increased when repeated postal reminders, e-mails or telephone calls were used [10]. Our findings are also consistent with previous findings showing lower response rates for each round of a reminding [16, 28, 29]. Further, our finding are in line with a previous study by Breen et al. which concluded that even though reminder phone calls increased response rates, this was not justified by the resources required [30]. If the purpose is to increase the population of patients with follow-up data, increasing the initial sample size and allowing a higher drop-out rate can be a feasible alternative to telephone reminders, as this is less time-consuming for researchers.Several studies have investigated the use of different types of reminders and incentives to complete surveys, with emails or text reminders being considered the most effective in terms of time consumption for researchers (as compared to telephone reminders). Still, a recent review of strategies to improve response rates concluded,that “research studies are needed to explore whether the different strategies used by researchers with the intent to improve response rates are acceptable to potential participants and to evaluate the potential synergistic effect of combinations of several strategies identified in this review.” [31].

### Baseline characteristics and outcomes

Reminder procedures are justified by the assumption that patients who do not respond to initial requests have different baseline characteristics and show different outcomes compared to patients who do respond to initial requests. To avoid biased estimates and increase internal validity, these multiple reminder procedures therefore seem justified [6, 7]. However, in this study, patients who responded after a telephone reminder did not differ from patients who responded without the telephone reminder in their baseline characteristics. The telephone reminder thereby only resulted in an increased response rate, while patients differing in baseline characteristics continued to be non-respondents. In other words, applying telephone reminders did not increase internal validity and, thereby, did not reduce the risk of selection bias [32]. A supplementary analysis was performed to investigate whether the initial respondents, i.e. those responding to the questionnaire without any reminders at all (*N* = 439), differed from patients responding following reminders. This was not the case, neither in terms of baseline characteristics nor changes in outcome estimates (data not shown).

Further, no differences were found in the outcome estimate between the two reminder groups, which is in line with findings from health survey studies on the general population [16, 19]. A study by Lall et al. used telephone interviews to collect follow-up information from non-responders in a cohort of patients with subacute and chronic LBP and found that non-responders had less favourable improvement in outcomes regarding back pain, disability and general health after 12 months [33]. In previous research, higher depression scores were associated with a less favourable functional improvement outcome in patients with LBP [34], and unemployment has been identified as the main predictor of disabling pain in patients with LBP [35]. While patients who respond to written or telephone reminders show similar outcomes, it seems reasonable to believe that the outcomes were different among patients who, regardless of reminder procedures, remain non-responders.

Combined with ethical concerns to not disturb research participants more than necessary, the degree to which reminders are employed must be thoughtfully considered, and our findings do not justify the use of telephone reminders in the present population, considering the outcome estimate [32].

### Strengths and weaknesses

A strength of our study is our use of validated questionnaires, increasing the possibilities of comparing our findings with results from other studies. Another strength of this study is the stringent reminder procedure, where the same researcher (AR) performed all the telephone reminder calls, to ensure a consistent approach in the reminder procedure.

Patients were recruited from a spine clinic at a public hospital with an uptake area covering the Central Denmark Region, corresponding to a population of 1.3 million Danes. Access to public hospitals is equal for all and free of charge in Denmark, and we therefore believe that the findings are generalisable to the Danish and European populations with chronic LBP. This is an ancillary trial using data from a prospective study, which is a practical and ethical approach; however, it is a limitation of the design. Collecting outcomes at more time-points could provide more information. It is possible that changes in outcomes were present after three or 6 months, however we lack the information to ascertain this. This study is examining response to online questionnaires and results may differ for paper-based questionnaires. The two different methods may appeal to different populations of potential respondents with different sociodemographic, behavioral, and health characteristics [36, 37]. Generalizability of this study may therefore be limited to studies using online questionnaires only. However, studies show, that patient-reported outcomes collected through electronic questionnaires are comparable to those collected via paper questionnaires. In accordance with research in this field, the electronic versions of the questionnaires were designed to be visually comparable to the original paper versions. For these reasons, we believe that results were not affected by the fact that a small proportion of patients (*N* = 29) replied paper versions of the questionnaire [38, 39].

### Implications for practice

Based on our findings, researchers should consider how time and resources in studies using patient-reported questionnaires is best spent. By far the most time consuming part of our data collection was the time spent on reaching non-responders by phone, and as seen in the results, using telephone-responders to increase response-rates did not change the findings on the patient reported outcomes or resulted in reaching participants with different baseline characteristics. Instead, it was evident; that patients not responding at all were systematically different from responders, indicating that missing was *not* at random. This further leads to the consideration, that imputation of missing data in surveys like these could lead to bias of findings, and therefore should not be performed [40]. Rather, researchers using patient-reported outcomes issued through surveys should focus on developing data collection methods to include the target groups that are typically hard to reach.

## Conclusions

Using a telephone reminder had no consequence on outcome estimates nor were there any differences in baseline characteristics between patients who responded before or after the telephone reminder.

## Data Availability

The datasets generated and/or analysed during the current study are not publicly available due to limitations of ethical approval involving the patient data and anonymity but are available from the corresponding author on reasonable request.

## Abbreviations

IQR: Interquartile Range
LBP: Low Back Pain
MDI: Major Depression Inventory
MRI: Magnetic Resonance Imaging
RMDQ: Roland Morris Disability Questionnaire
SD: Standard Deviation
